# Ovarian fibrosis: molecular mechanisms and potential therapeutic targets

**DOI:** 10.1186/s13048-024-01448-7

**Published:** 2024-07-05

**Authors:** Mengqing Gu, Yibo Wang, Yang Yu

**Affiliations:** 1 State Key Laboratory of Female Fertility Promotion, Center for Reproductive Medicine, Department of Obstetrics and Gynecology, Peking University Third Hospital, Beijing, 100191 China; 2 Key Laboratory of Assisted Reproduction (Peking University), Beijing Key Laboratory of Reproductive Endocrinology and Assisted Reproductive Technology, Ministry of Education, Beijing, 100191 China; 3 Clinical Stem Cell Research Center, Peking University Third Hospital, Beijing, 100191 China; 4 Beijing Key Laboratory of Reproductive Endocrinology and Assisted Reproductive Technology, Beijing, 100191 China; 5 Institute of Obstetrics and Gynecology, The Third Affiliated Hospital of Guangzhou Medical University, Guangzhou, 510150 China

**Keywords:** Ovarian fibrosis, Ovarian dysfunction, Ovarian aging, Assisted Reproductive Technology (ART), Therapeutic targets

## Abstract

Ovarian fibrosis, characterized by the excessive proliferation of ovarian fibroblasts and the accumulation of extracellular matrix (ECM), serves as one of the primary causes of ovarian dysfunction. Despite the critical role of ovarian fibrosis in maintaining the normal physiological function of the mammalian ovaries, research on this condition has been greatly underestimated, which leads to a lack of clinical treatment options for ovarian dysfunction caused by fibrosis. This review synthesizes recent research on the molecular mechanisms of ovarian fibrosis, encompassing TGF-β, extracellular matrix, inflammation, and other profibrotic factors contributing to abnormal ovarian fibrosis. Additionally, we summarize current treatment approaches for ovarian dysfunction targeting ovarian fibrosis, including antifibrotic drugs, stem cell transplantation, and exosomal therapies. The purpose of this review is to summarize the research progress on ovarian fibrosis and to propose potential therapeutic strategies targeting ovarian fibrosis for the treatment of ovarian dysfunction.

## Introduction

The Ovary is one of the most important reproductive organ in mammalian species, which plays crucial roles in maintaining female fertility and regulating the systemic health of the body [1]. As the female gonads, the ovaries produce mature eggs and undergo ovulation after the reproductive system matures during puberty [2]. The hormones secreted by the ovaries mainly consist of estrogen and progesterone, with a small amount of androgen also being secreted. Under the influence of these hormones, the endometrium undergoes cyclic changes to ensure a normal menstrual cycle [3]. Damage to the ovaries can significantly impact ovarian function, leading to a reduction in both the quantity and quality of eggs, as well as endocrine abnormalities. Furthermore, abnormalities in the ovaries are closely associated with the development of various gynecological diseases in women, such as polycystic ovary syndrome (PCOS), premature ovarian failure (POF), endometriosis, and other ovarian dysfunction and aging caused by abnormal ovarian fibrosis. These diseases affect women’s fertility and can lead to other health issues. Therefore, a deeper understanding of the molecular mechanisms behind pathological conditions such as ovarian fibrosis is of great significance for the development of new treatment methods and the improvement of women’s health [4–7]. In this paper, our focus is specifically on examining the impact of abnormal ovarian fibrosis on ovarian dysfunction and ovarian aging exploring potential interventions.

Fibrosis, characterized by excessive fibroblast proliferation and extra-cellular matrix deposition, can occur in all organs and tissues. Excessive fibrosis potentially leading to organ dysfunction and death [4]. Inflammation, signaling pathways, metabolic homeostasis, and the extracellular matrix (ECM) are key triggers of fibrosis, leading to the activation of fibroblasts and ECM deposition [5]. These factors may regulate ovarian fibrosis by coordinating fibroblast proliferation and extracellular matrix deposition in the ovary [6].

Present research on fibrosis mechanisms and treatments predominantly focuses on liver, renal, pulmonary, and cardiac fibrosis [7–10]. However, fewer studies have addressed ovarian fibrosis, despite the ovary’s crucial role in egg storage, ovulation, and reproductive hormone secretion, which significantly impacts female fertility [11]. Abnormal ovarian fibrosis, a chronic and progressive condition, is influenced by various factors such as inflammation, aging, mechanical damage, PCOS [12], premature ovarian insufficiency (POI) [13], and ovarian endometriotic cysts [6]. Exacerbation of ovarian fibrosis is one of the signs of ovarian function decline and aging in women [14]. Recently, many researchers have raised their focus on ovarian fibrosis and verified that targeting ovarian fibrosis can significantly rescue ovarian dysfunction and prevent ovarian aging [15–18], which remind us that the important role of ovarian fibrosis and provide potential therapeutic targets for improving the success rate in clinical assisted reproductive technology (ART) for women.

In this paper, we elucidate the key characteristics and mechanisms underlying fibrosis. This review synthesizes prior research on fibrosis pathogenesis and outlines current effective treatments for fibrosis-related diseases. Additionally, this review encapsulates effective clinical treatments for ovarian fibrosis, aiming to offer novel insights for its management.

## Methods

The study is a narrative review. We searched the published articles in PubMed database, containing key words Fibrosis, Ovarian fibrosis, PCOS, TGF-β, ECM, Inflammation, fibrosis therapeutic, Antifibrosis drugs, Stem cells and exosomes, Ovarian fibrosis clinical diagnosis, POF, Ovarian aging, Fertility, ovarian endometriosis cysts, Ovarian Reserve, Infertility, Mechanisms of ovarian fibrosis in the English-language literature until April 2024.

## Ovarian fibrosis: molecular mechanisms

### Ovarian fibrosis: inducers

#### Transforming growth factor beta (TGF-β)

Transforming growth factor beta (TGF-β), including three isoforms (TGF-β1, TGF-β2, and TGF-β3), plays a crucial role in cell differentiation, migration, proliferation, and gene expression [19–23]. The active form of TGF-β binds to TGF-β receptor 2 (TGFR2), which subsequently recruits and activates TGFR1, triggering downstream Smad signaling pathways [29]. The TGF-β signaling pathway has multiple functions in the mammalian ovary. It regulates the recruitment of primordial follicles and the FSH sensitivity of growing follicles in an inhibitory manner [24, 25]. In addition, TGF-β is able to regulate follicular development, ovulation and COC expansion, and luteinisation after ovulation through its downstream Smad signaling pathway [26–28]. Abnormally elevated levels of TGF-β1 in the ovary can lead to follicular dysplasia and ovulation failure [29]. TGF-β is pivotal in tissue fibrosis, mediating this process through classical Smad-dependent or non-Smad pathways [30–32]. Overexpression of TGF-β induces epithelial-mesenchymal transition (EMT) and extracellular matrix (ECM) deposition, contributing to the onset of fibrotic diseases like lung, kidney, and liver fibrosis [33]. In the vasculature, endothelial cells, mural cells, and epithelial cells are primary targets of TGF-β. Endothelial and mural cells foster fibrosis by expressing ECM proteins or secreting fibroblast activating factors in response to TGF-β [32]. Endothelin (ET-1), a form of angiotensin, indirectly stimulates fibrogenesis by enhancing TGF-β1-induced EMT and the expression of pro-fibrotic genes and proteins [34].

#### Extracellular matrix(ECM)

The ECM is a three-dimensional, non-cellular macromolecular network comprising collagen, proteoglycans, elastin, fibronectin, laminin, and various glycoproteins [35]. Present in all tissues, the ECM is a highly dynamic structure that undergoes continual and controlled remodeling. By interacting with cells, the ECM regulates diverse functions such as proliferation, migration, and differentiation [36]. Matrix metalloproteinases (MMPs) are the principal enzymes involved in ECM degradation, remodeling the matrix by degrading collagen [37]. MMP regulation involves several kinases, including mitogen-activated protein kinase (MAPK), p38, focal adhesion kinase (FAK), extracellular signal-regulated kinase (ERK)1, and protein kinase B (Akt), which induce MMP transcription and translation through phosphorylation and modification of cellular signaling [36, 38, 39]. Besides MMPs, various proteases such as ADAM, ADAMTS, fibrinolytic enzymes, histones, and neutrophil elastase also regulate ECM remodeling [40]. The extracellular matrix is essential for follicular growth and functional maturation [41]. However, the excessive deposition of ECM may lead to ovarian fibrosis and functional impairment [42, 43]. Research has revealed that platelet endothelial aggregation receptor 1 in fibroblasts promotes lung fibrosis by regulating the proliferation of activated fibroblasts and ECM deposition [44]. The accumulation of extracellular matrix (ECM) increases progressively during follicular development and ovary aging. ECM plays a crucial role in promoting fibrosis, making it a key contributing factor in the development of abnormal fibrosis in the ovary [45]. Therefore, the elevated ECM levels in the ovary exacerbate the progression of aberrant fibrosis.

#### Connective tissue growth factor (CTGF)

CTGF, a multifunctional protein belonging to the CCN family, regulates diverse biological processes, including cell proliferation, differentiation, and adhesion. CTGF is necessary for mammalian follicular growth and development as well as ovulation [46]. CTGF is secreted in the ovary by follicular granulosa cells and fibroblasts via autocrine or paracrine pathways [47]. CTGF participates in disease-related pathways including the Hippo pathway, p53, and the nuclear factor κB (NF-κB) pathway. Additionally, it functions as a downstream effector in the development of diseases like inflammation, fibrosis, and cancer [48]. Furthermore, CTGF fosters fibrogenesis through interactions with the Slit2/Robo signaling pathway and competitive binding of miR-18a with the lncRNA PFAL [49, 50].

### Inflammation

Tissue injury and inflammation serve as crucial triggers for fibrosis. Tissue injury induces general inflammation and also triggers diverse inflammatory responses by recruiting and activating various cells from both the innate and adaptive immune systems [51]. Studies have shown that T cells and macrophages, through regulating reactive oxygen species, protease-activated receptors, and pro-fibrotic cytokines, contribute to lung fibrosis via the TGF-β and PDGF signaling pathways [52]. Some studies have reported that fibroblasts exacerbate ovarian fibrosis during ovarian aging through the modulation of inflammation [53]. Sustained elevation of IL-6 and IL-8 can provoke an inflammatory response and fiber formation around the ovaries, potentially leading to ovulation failure [6].

## Ovarian fibrosis: inhibitors

### Bone morphogenetic protein 7 (BMP-7)

Bone morphogenetic protein 7 (BMP-7) is part of the bone morphogenetic proteins (BMPs) superfamily, with BMP signaling reliant on ligand binding to target cells, particularly type I and type II BMP receptors. The SMAD pathway represents the classical downstream signaling cascade of the BMPs superfamily [54]. BMP-7 promotes the expression of FSH and LH receptors in human ovarian granulosa cells, thereby promoting normal follicular development prior to ovulation and luteinization after ovulation [55, 56].BMP-7 could alleviate cardiac fibrosis by inhibiting EMT [57]. Furthermore, BMP-7 ameliorates diabetes-induced kidney fibrosis by upregulating Id2 via the MAPK signaling pathway and downregulating miR-21 [58, 59]. BMP-7 may also inhibit pulmonary fibrosis by activating the BMP-7/Smad pathway and concurrently inhibiting the TGF-β/Smad pathway [60]. BMP4 and its receptor are widely expressed in the ovary, and dysregulation of BMP4 expression may play an important role in follicular development and polycystic ovary syndrome (PCOS), which has a strong association with ovarian fibrosis [61].

### Peroxisome proliferator-activated receptor gamma(PPAR-γ)

PPARγ, belonging to the PPAR family with isoforms PPARα, PPARδ, and PPARγ, plays a pivotal role in gene expression regulation. They are involved in lipid storage and mobilization, glucose metabolism, morphogenesis, and inflammatory responses [62]. PPARγ is predominantly expressed in small sinus follicles, with low expression in the corpus luteum and large follicles [63]. Studies have shown that piperine attenuates pathological myocardial fibrosis via the PPAR-γ/AKT signaling pathway [64]. Additionally, KLF14 has been found to inhibit hepatic fibrosis by targeting PPARγ [65]. Furthermore, PPAR-γ is an agonist of rosiglitazone, and rosiglitazone is able to reduce the expression of the fibrosis-promoting factor TGF-β in ovarian granulosa cells, so this suggests that PPAR-γ has a positive effect on improving ovarian fibrosis [66–68].

### Vascular endothelial growth factor (VEGF)

**Fig. 1:**
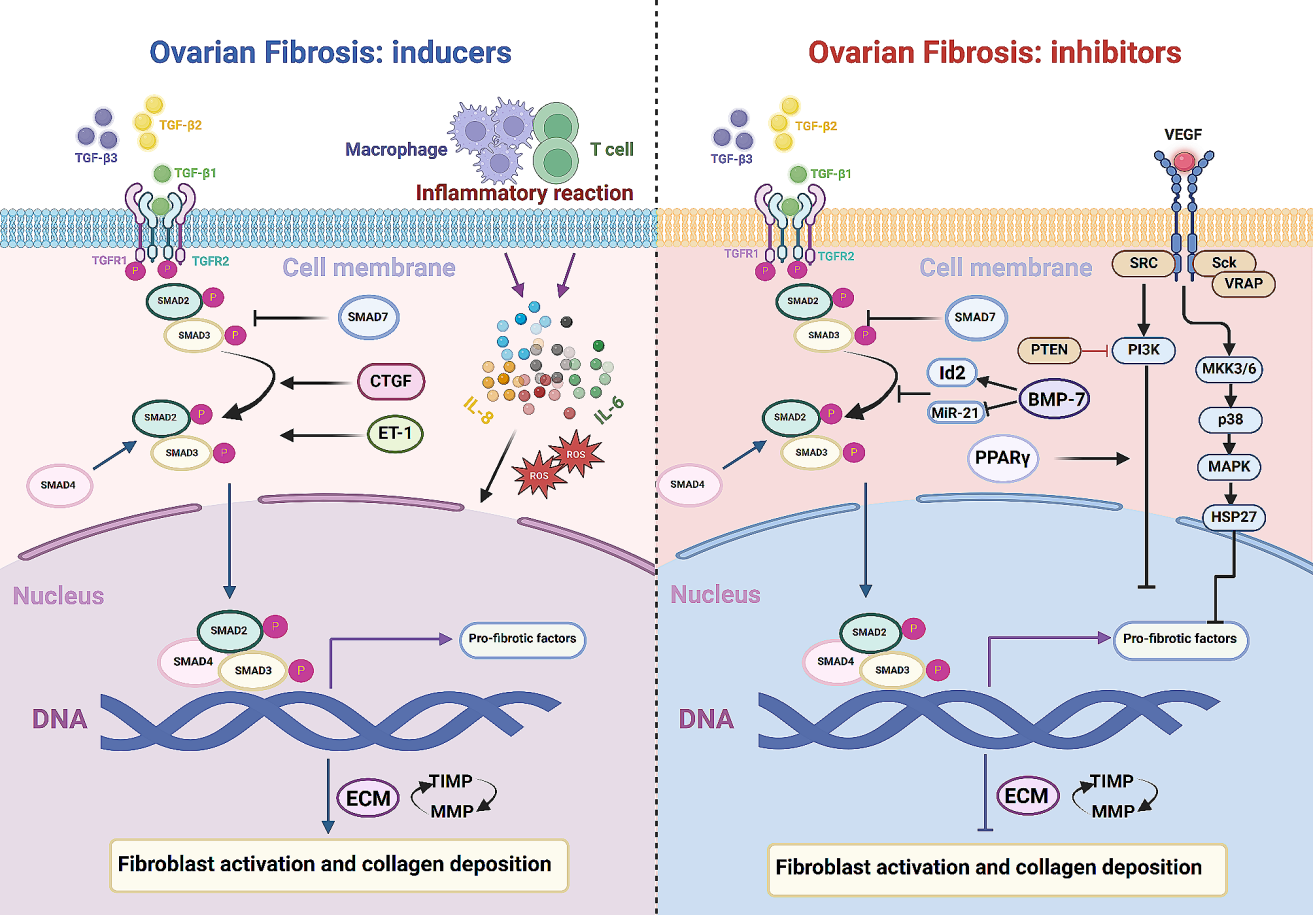
Mechanisms of ovarian fibrosis

VEGF is mainly expressed in endothelial cells in blood vessels, which are components of the inner wall of blood vessels [69]. These cells play a key role in vascular function, both as a barrier between blood and tissue and as endocrine organs that actively regulate vasodilatation/constriction, immune responses, angiogenesis and coagulation/fibrinolysis [70]. In terms of promoting fibrosis, endothelial cells establish a vicious cycle of pro-fibrotic activity by activating myofibroblasts to produce excessive amounts of ECM [71]. VEGF comprises six members: VEGF-A, VEGF-B, VEGF-C, VEGF-D, VEGF-E, and PlGF, signaling through tyrosine kinase receptors VEGF receptor 1 (VEGFR1) and VEGF receptor 2 (VEGFR2), along with the co-receptor neuropilin. VEGF is crucial in regulating angiogenesis and associated diseases, predominantly through VEGF-A [72, 73]. VEGF is closely associated with fibrosis and has been demonstrated to inhibit lung fibrosis [74]. The microRNA-21 [75] and Pi3k/Akt pathways [76] induce VEGF expression, which in turn ameliorates liver and oral submucosal fibrosis. VEGF is widely expressed in the ovary and affects follicular development, luteal function and thus fertility by regulating angiogenesis [77], suggesting that VEGF may have a role in ameliorating ovarian fibrosis (Fig. 1).

TGF-β is pivotal in tissue fibrosis, mediating this process through classical Smad-dependent or non-Smad pathways. The active form of TGF-β binds to TGFR2, which then recruits and activates TGFR1, triggering downstream signaling pathways. Upon activation, TGFR1 phosphorylates SMAD2, SMAD3, and SMAD4. SMAD7 competes with TGFR1 for interaction with SMAD proteins, thereby inhibiting their activation and signal propagation. The activated SMAD proteins dissociate from TGFR1 and form a complex with SMAD4. This complex then translocates to the nucleus to regulate the transcription of target genes. TGF-β can regulate ECM. Overexpression of TGF-β induces fibroblast activation and collagen deposition, contributing to the onset of fibrotic diseases. Inflammation, CTGF, and ET-1 enhance TGF-β’s function, promoting fibrogenesis. BMP-7 may inhibit fibrosis by antagonizing the TGF-β/Smad pathway. VEGF pathway, closely associated with fibrosis, has been shown to inhibit fibrosis. PPAR-γ could enhance the VEGF pathway to inhibit fibrosis. TGFR1:TGF-β receptor 1, TGFR2: TGF-β receptor 2, ECM: Extracellular matrix, CTGF: Connective tissue growth factor, ET-1: Endothelin, PPAR-γ: Peroxisome proliferator-activated receptor gamma, BMP-7:Bone morphogenetic protein 7, VEGF: Vascular endothelial growth factor, MMP: Matrix metalloproteinases, TIMP: Tissue inhibitors of metalloproteinases.

### Targeting therapeutic in fibrosis

The targeting of therapeutics in fibrosis focuses on various organs such as the kidney, liver, lung, and heart, with treatments predominantly aimed at mitigating organ injury, inhibiting inflammation and fibroblast activation, and regulating extracellular matrix deposition [5]. Chronic liver injuries, for example, lead to liver inflammation and fibrosis, with activated hepatic myofibroblasts secreting extracellular matrix proteins and forming fibrous scarring. Understanding liver fibrosis mechanisms aids in identifying novel therapeutic targets [9]. Therapies such as pan-caspase inhibitors (Emricasan, VX-166), PPARδ agonists [78, 79], IL-22 [80], Serum amyloid P (SAP) [81], vascular adhesion protein 1 (VAP1) [82], lysophospholipids (LPA), and TGFβ inhibitors [83] all contributed to the amelioration of liver fibrosis by modulating fibroblast activation and ECM deposition.

Pulmonary fibrosis, a progressive lung disease, is characterized by the proliferation of fibroblasts and remodeling of the extracellular matrix (ECM), leading to irreversible lung structure damage and ultimately resulting in mortality [84]. Many factors are involved in the development of pulmonary fibrosis including transforming growth factor-beta (TGF-β), vascular endothelial growth factor (VEGF), platelet-derived growth factor (PDGF), fibroblast growth factor (FGF), and hepatocyte growth factor (HGF) [85]. Nintedanib and pirfenidone are commonly prescribed treatments for pulmonary fibrosis [86]. Additionally, stem cell transplantation is emerging as a significant therapeutic approach in pulmonary fibrosis treatment [87]. Furthermore, inhibiting the expression of fibrogenesis-associated molecules, including TGF-β, FGF, CTGF, and CD44/HA, is instrumental in preventing fibrogenesis [88].

Cardiac fibrosis manifests in various heart diseases such as myocardial infarction, hypertrophic cardiomyopathy, dilated cardiomyopathy, diabetic cardiomyopathy, and aortic valve stenosis [89]. Following cardiac damage, cardiac fibroblasts differentiate into myofibroblasts, promoting extracellular matrix (ECM) and collagen deposition. The sustained proliferative activation of myofibroblasts results in fibrotic scarring and cardiac dysfunction [90]. Research on cardiac fibrosis treatment encompasses fibroblast reprogramming, CAR-T cell-based immunotherapy, and stem cell therapies, including the use of extracellular vehicles (EVs), secretomes, exosomes, proteins, and drugs like atorvastatin and enrasentan have shown fibrosis attenuation in clinical studies [91, 92]. MicroRNAs (miRNAs) represent a potential avenue for reprogramming, as they can enhance the expression of transcription factors like GMT and Nkx2.5. However, the clinical translation of novel mediators like miRNAs and cytokines for attenuating cardiac fibrosis is still in progress.

**Fig. 2:**
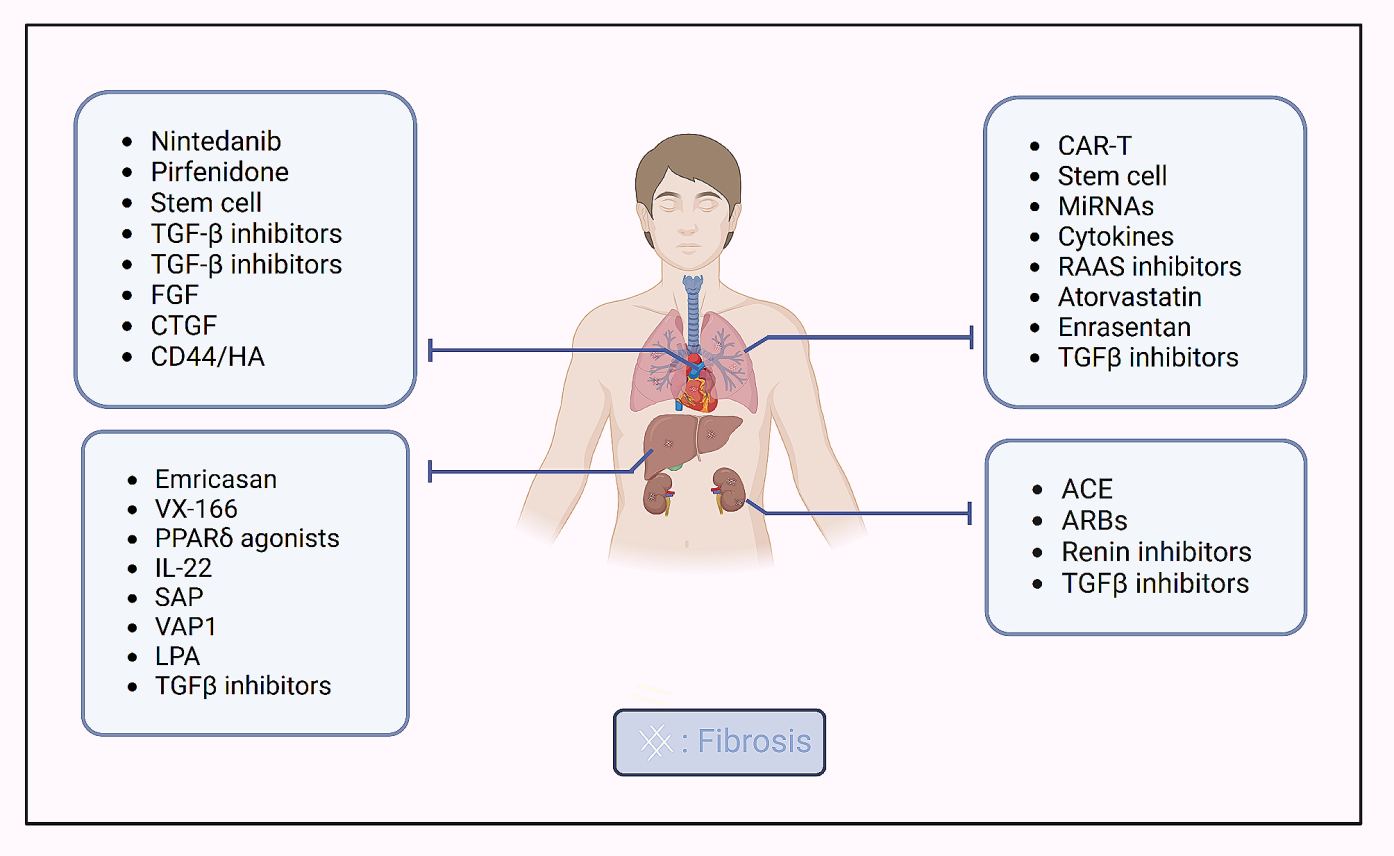
Targeting therapeutic in fibrosis

Kidney fibrosis, encompassing tubulointerstitial fibrosis, tubular atrophy, and glomerulosclerosis, commonly underlies chronic kidney disease. Renal fibrosis is primarily characterized by excessive extracellular matrix deposition and chronic inflammation [93]. Currently, commercially available treatments for renal fibrosis include angiotensin-converting enzyme (ACE) inhibitors, angiotensin II receptor blockers (ARBs), and renin inhibitors [8] (Fig. 2).

This figure illustrates the range of therapeutic agents and their targets in the treatment of fibrosis across various organs including kidney, liver, lung, and Cardiac. Emricasan and VX-166: an-caspase inhibitors, SAP: Serum amyloid P, VAP1: Vascular adhesion protein 1, LPA: Lysophospholipids, PDGF: Platelet-derived growth factor, FGF: Fibroblast growth factor, HGF: Hepatocyte growth factor, ACE: Angiotensin-converting enzyme, ARBs: Angiotensin II receptor blockers, RAAS: Renin-angiotensin-aldosterone system.

### Current investigations of ovarian fibrosis

#### Polycystic ovary syndrome (PCOS)

Polycystic ovary syndrome (PCOS) is a complex, heterogeneous endocrine disorder predominantly affecting women of childbearing age, with an incidence rate of approximately 10–15%. Clinical manifestations of PCOS often include insulin resistance, elevated androgen levels, anovulation or oligoovulation, and the presence of cystic follicles [94]. Ovarian fibrosis is a major cause of ovarian hypoplasia and ovarian decline. The ovaries of PCOS patients express abnormally high levels of TGF- β1. TGF- β1 promotes the production of ECM in mesenchymal cells and the production of enzymes that impede ECM breakdown. Finally, excessive ECM accumulation in the ovaries of PCOS patients promotes ovarian interstitial fibrosis, endangering women’s reproductive health and quality of life [95]. Additionally, Hyperandrogenism, a hallmark of PCOS, is linked to the promotion of fibrogenic gene transcription and regulation of epithelial-mesenchymal transition (EMT) via the TGF-β signaling pathway, as evidenced in studies using the androgen analog dehydroepiandrosterone in PCOS rat models to induce ovarian fibrosis [96]. Moreover, hyperandrogenism stimulates chronic ovarian inflammation and activates the NLRP3 inflammasome, further contributing to the onset of ovarian fibrosis [97].

### Premature ovarian failure (POF)

Premature ovarian failure (POF) is characterized by diminished development of mature follicles and an increased prevalence of atretic follicles and sensitivity to gonadotropins (Gn) is low in the ovaries [98]. Another feature of POF is follicular depletion, which is characterized by the filling of the ovarian cortex or interstitium with fibrous tissue, a thickening of the ovarian envelope and a reduction in the number of follicles [99]. Ovarian fibrosis resulting from POF is closely associated with reduced vascularity in the ovarian interstitial tissue. Transplantation of human amniotic epithelial cells has been found to inhibit apoptosis in ovarian granulosa cells, activate the TGF-β/Smad signaling pathway, and enhance the restoration of vascularity and functionality in damaged ovaries, thereby improving ovarian fibrosis symptoms [100].

### Ovarian aging

Fibrosis is a chronic healing response characterized by excessive formation and deposition of extracellular matrix (ECM), chronic inflammatory, and reduction in parenchymal cells [101]. As fibrosis develops and progresses, it affects nearly all tissues and organs, resulting in increased morbidity and mortality associated with aging-related diseases worldwide [4]. The ovary is the first reproductive organ to age in the body, and aging leads to abnormalities in ovarian oxidative stress, inflammation, and mitochondrial dysfunction, thereby promoting the development of ovarian fibrosis [102]. Some studies have reported that fibroblasts exacerbate ovarian fibrosis during ovarian aging through the modulation of inflammation [53]. It has been demonstrated that the antifibrotic drugs pirfenidone and, BGP-15 as well as metformin can ameliorate fibrosis and thereby alleviate fertility in senescent mice by eliminating fibrotic collagen and modulating the immune microenvironment, respectively [17, 18]. Ovarian fibrosis severely affects ovarian function, causing ovarian failure and hormonal abnormalities that reduce female fertility [14]. With the worldwide increase in the average age of childbearing, preventing and improving age-related infertility becomes crucial [103]. Therefore, targeting the improvement of age-related ovarian fibrosis could be a potential therapeutic approach to preserving ovarian function and enhancing female fertility.

### Post-radiotherapy complications and surgeries

Radiation therapy, a common cancer treatment, eradicates cancer cells by inducing DNA damage. The ovaries are particularly susceptible to radiation damage due to their inherent properties [104]. Radiation exposure typically results in ovarian tissue atrophy and fibrosis, with antifibrotic drugs showing potential in ameliorating radiation-induced ovarian fibrosis and aiding in the restoration of ovarian function [105]. Moreover, mechanical damage incurred during certain surgical procedures, such as abortions, ovarian cyst removal, and surgical interventions for PCOS, can directly or indirectly lead to the development of ovarian fibrosis [106].

### Ovarian chocolate cysts

**Fig. 3:**
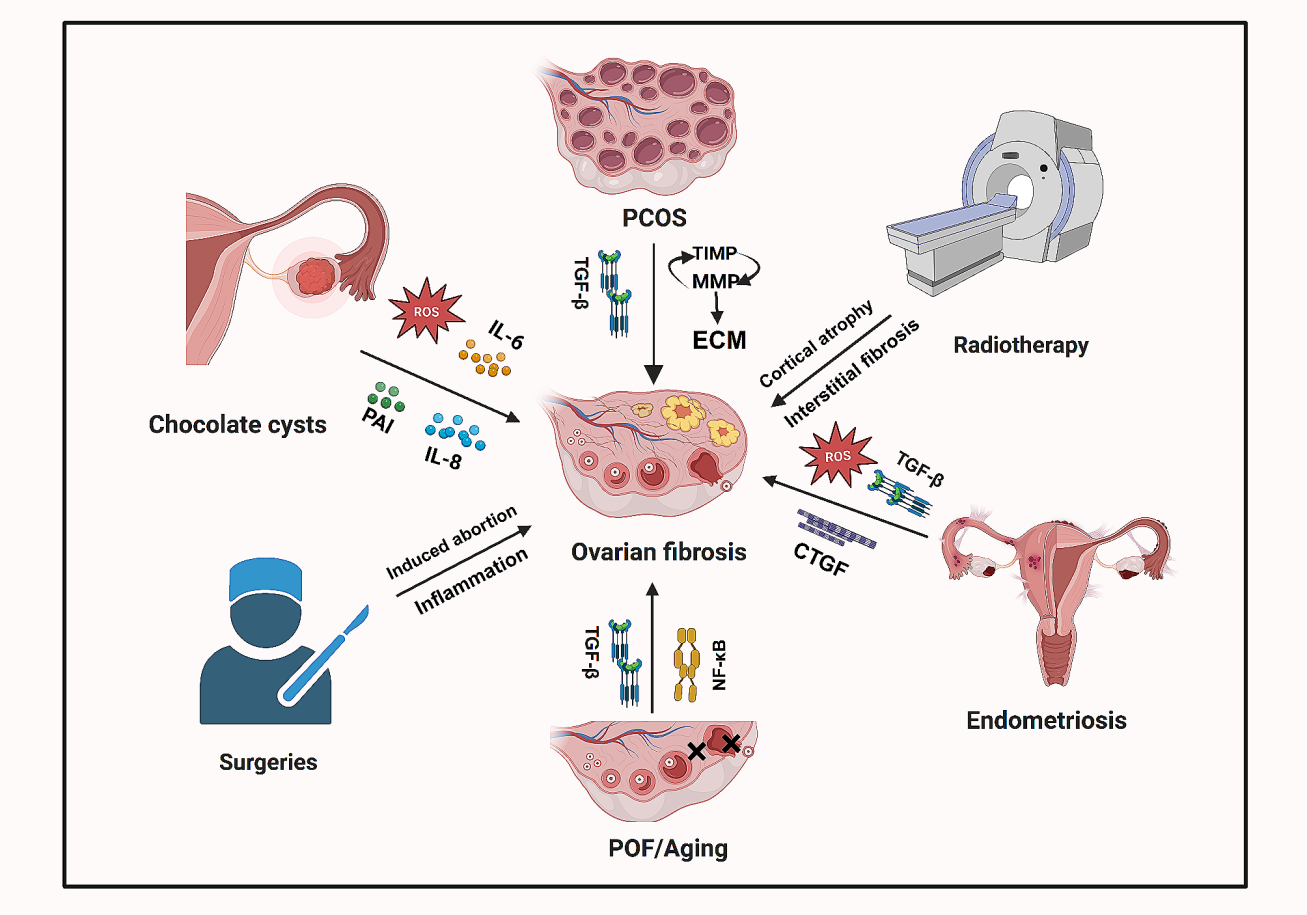
Female gynecological associated with ovarian fibrosis

Ovarian chocolate cysts are characterized primarily by smooth muscle metaplasia and fibrosis. These cysts typically develop in a unilateral ovary [107]. Ovarian chocolate cysts show marked cortical fibrosis and absence of hair follicles which in turn affects ovarian angiogenesis, ovulation, and hormonal disorders thereby impairing ovarian function [108]. They are strongly linked to an inflammatory response marked by the secretion of IL-6 and IL-8, resulting in localized ovarian inflammation and fibrosis [109]. Additionally, these cysts are associated with elevated levels of reactive oxygen species and abnormal expression of the fibrinogen activating system, further promoting fibrosis development [6]. Radiation exposure typically results in ovarian tissue atrophy and fibrosis, with antifibrotic drugs showing potential in ameliorating radiation-induced ovarian fibrosis and aiding in the restoration of ovarian function [105] (Fig. 3).

This figure provides an integrative overview of various gynecological conditions contributing to the development of ovarian fibrosis. It illustrates the release of ROS, IL-6, PAI, and IL-8 in chocolate cysts, implicated in the pathogenesis of ovarian fibrosis. Ovarian fibrosis resulting from POF is closely associated with reduced vascularity in the ovarian interstitial tissue and is mediated through TGF-β and NF-κB signaling pathways. PCOS also contributes to ovarian fibrosis by dysregulating the ECM and TGF-β pathway. Endometriosis, radiotherapy, and surgical interventions are additional factors that can lead to ovarian fibrosis. ROS: Reactive oxygen species, IL-6: Interleukin-6, PAI: Plasminogen activator inhibitor, IL-8: Interleukin-8, POF: Premature ovarian failure, PCOS: Polycystic ovary syndrome.

### Potential clinical therapeutic methods of ovarian fibrosis

#### Antifibrosis drugs

Ovarian fibrosis impairs ovarian function, inhibiting oocyte growth and ovulation onset, thereby severely affecting female fertility. Pirfenidone, an anti-inflammatory antioxidant, counteracts fibrosis by inhibiting TGFβ1, as evidenced in lung studies [110]. Studies report that antifibrotic drugs pirfenidone and BGP-15 promote ovulation and enhance fertility by reducing fibrillar collagen accumulation in mouse ovaries [18]. Metformin, commonly used for diabetes treatment, has been found to prevent age-related ovarian fibrosis by modulating immune cell and fibroblast populations [17]. Rhamnocitrin has been shown to increase PPAR-γ activity and inhibit the TGF-β1/Smad pathway in the ovaries of PCOS rats, ameliorating ovarian fibrosis [111]. The protein p66Shc, a member of the ShcA family, may be targeted by Sirt1 to induce reactive oxygen species (ROS) and promote fibrosis. Consequently, inhibiting p66Shc could be a strategy to prevent hyperandrogenism-induced ovarian fibrosis [112]. Rosiglitazone may delay fibrosis onset by reducing levels of TGF-β1 and CTGF [113]. The BRD4 and HIF-1α induced binding to the histone 3/4 acetylation-modified AR promoter, while the BRD4-selective inhibitor JQ1 decreased this binding, ameliorating adverse expressions in PCOS ovarian and DHEA-treated granulosa cells [12]. Although the causes of ovarian fibrosis are varied, no drugs have been significantly effective in treating it.

### Stem cells and exosomes for the treatment of ovarian fibrosis

Premature ovarian failure (POF) is primarily characterized by ovarian fibrosis and impaired follicular development [114]. Studies suggest that in POF rat models, transplantation of human umbilical cord mesenchymal stem cells (hUMSCs) inhibits ovarian fibrosis by regulating mesenchymal cell differentiation and repairing ovarian function via the TGF-β1/Smad3 and AMPK/NR4A1 signaling pathways [13, 115]. Additionally, menstrual blood-derived CD146 + mesenchymal stem cells have been shown to ameliorate ovarian fibrosis and increase the birth rate in POF rat models by targeting the TGF-β1/Smad3 signaling pathway [99]. Stem cell exosomes are cellular secretions derived from stem cells. These exosomes carry biologically active substances, including proteins, mRNA, and microRNA, from stem cells. They function to reduce apoptosis, attenuate inflammatory responses, and promote angiogenesis [116]. A study reported that stem cell-derived exosomes, particularly those containing miR-130a, significantly ameliorated fibrosis [117]. Additionally, miR-214 in endometrial mesenchymal stromal cell-derived exosomes inhibited endometriosis-related fibrosis [118]. Human umbilical cord mesenchymal stem cell-derived exosomes (HUCMSC-Exos) improved the ovarian microenvironment in POF rat models by engaging in signaling pathways associated with immune regulation, cellular viability, inflammatory control, fibrosis, and metabolism [119]. Stem cells and their secretions show considerable potential in improving ovarian fibrosis and enhancing female fertility.

#### Surgery for pathological ovarian fibrosis

**Fig. 4:**
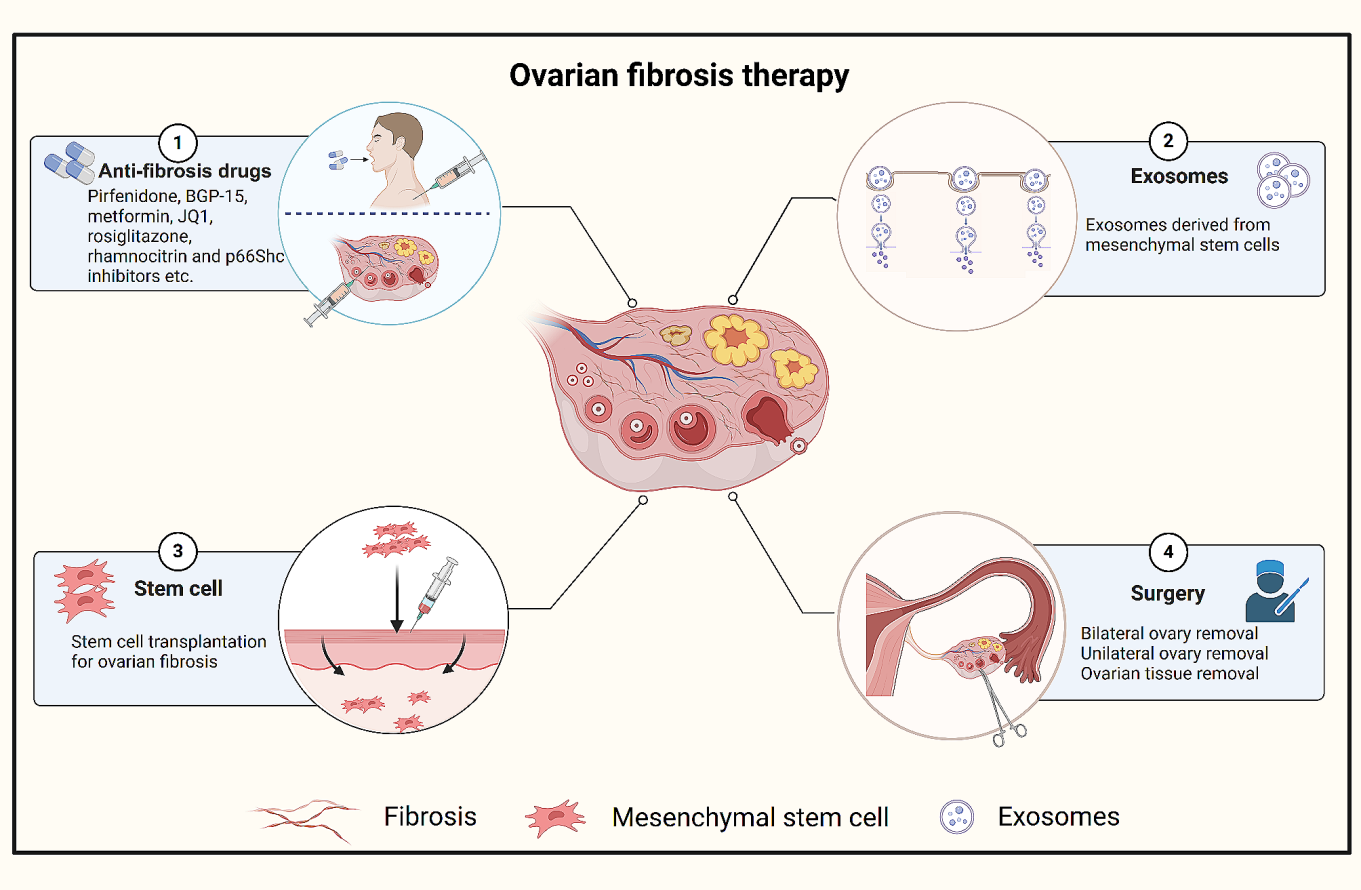
Potential therapy strategies for ovarian fibrosis

During early ovarian fibrosis, symptoms may be managed with medication and lifestyle modifications, whereas surgery may be necessary in more severe cases. Surgical intervention for ovarian fibrosis, primarily involving the removal of ovarian cysts, aims to alleviate symptoms and restore ovarian function. In cases of smaller ovarian cysts, cystic fluid drainage via puncture can provide symptom relief [120]. However, in severe cases of ovarian fibrosis leading to complete loss of ovarian function, total ovarian removal may be considered. In cases of partial ovarian tissue fibrosis, surgical removal of fibrotic tissue, while preserving normal tissue, can help maintain ovarian function [121] (Fig. 4).

Ovarian fibrosis impairs ovarian function, inhibiting oocyte growth and the onset of ovulation, thereby severely affecting female fertility. This figure illustrates the range of therapeutic agents and their targets in the treatment of ovarian fibrosis. Current treatments for ovarian fibrosis consist of medication, stem cell transplantation, stem cell secretory exosomes, and surgery. JQ1: BRD4-selective inhibitor, BGP-15: [O-[3-piperidin-2-hydroxy-1-propyl]-nicotine-amidoxime.

## Discussion

Ovarian fibrosis, characterized by fibrotic changes in ovarian tissue, may lead to impaired ovarian function, reducing the production and release of eggs from the ovaries and seriously affecting a woman’s fertility. The pathogenesis involves factors like TGF-β, ECM components, CTGF, BMP-7, PPAR-γ, VEGF, and inflammation. It is also closely related to aging and reproductive disorders including PCOS, POI, and ovarian cysts. As these disorders progress, they can lead to ovarian fibrosis, affecting ovarian function. Currently, no effective drug treatment specifically for ovarian fibrosis exists, with reliance primarily on anti-fibrotic inhibitors. Recently, stem cells and their secretory exosomes have emerged as promising therapeutic options for fibrosis.

Ovarian fibrosis is influenced by various factors and has been found to be strongly associated with numerous gynecological diseases. This paper aims to elucidate the key features and molecular mechanisms of ovarian fibrosis. Additionally, it provides a comprehensive summary of the current molecular pathways associated with ovarian fibrosis, which contribute to the development of various ovarian diseases. Furthermore, it reviews potential clinical treatments for ovarian fibrosis, encompassing anti-fibrotic drugs, stem cell therapy, and surgical interventions, in order to identify potential therapeutic targets and options for preserving women’s ovarian function and enhancing their fertility.

Since fibrosis is often characterized by the activation of multiple pro-fibrotic pathways, a multipronged approach may be required to mitigate the progression of fibrosis. Based on the current sequencing results of the human ovary, future research can focus on important regulatory genes involved in human ovarian fibrosis, effectively combining basic research with clinical application and translation. Current research on ovarian fibrosis has predominantly focused on animal models. Therefore, future research should prioritize developing new and improved preclinical models that more accurately replicate human ovarian fibrotic disease to investigate pathogenic mechanisms and effective therapeutic interventions.

## Conclusions

Ovarian fibrosis is influenced by various factors and has been found to be strongly associated with numerous gynecological diseases. However, the current research on ovarian fibrosis is not thorough enough and more research is needed to complement the pathogenic mechanisms of ovarian fibrosis and to find new therapeutic targets to improve ovarian fibrosis. Our review aims to raise people’s attention of ovarian fibrosis and provide potential clinical treatment strategies to rescue women’s ovarian dysfunction and prevent ovarian aging, which shed a light for improving the success rate in clinical assisted reproductive technology (ART) for women.

## Data Availability

No datasets were generated or analysed during the current study.
